# Loss of Potassium and Chloride Transport Changes PM-Induced Epithelial Dysfunction

**DOI:** 10.2147/JIR.S564139

**Published:** 2026-03-04

**Authors:** Sandra Jaworowska, Kamila Maliszewska-Olejniczak, Agnieszka Łukasiak, Jakub Hoser, Mirosław Zając, Piotr Bednarczyk

**Affiliations:** 1 Department of Physics and Biophysics, Institute of Biology, Warsaw University of Life Sciences – SGGW, Warsaw, Poland

**Keywords:** particulate matter, potassium and chloride transport, oxidative stress, inflammation, mitochondrial function, epithelial barrier integrity

## Abstract

**Background:**

Chronic exposure to particulate matter (PM) is recognized as a significant contributor to respiratory health complications, including oxidative stress, inflammatory responses, and compromised epithelial barrier function. In this work, we ask whether the transport of potassium and chloride through the large-conductance calcium-activated potassium (BK_Ca_) channel and the cystic fibrosis transmembrane conductance regulator (CFTR) channel may change PM-induced epithelial dysfunction.

**Methods:**

This study aimed to evaluate the impact of PM on cell variability, ROS level, inflammation, mitochondrial function, intracellular calcium homeostasis, and epithelial barrier integrity in three different airway epithelial cell lines: wild-type human bronchial epithelial cells (HBE WT), HBE WT cells with disruption of the *KCNMA1* gene encoding the α-subunit of the BK_Ca_ channel (HBE ΔαBK_Ca_) with lost potassium transport, and cystic fibrosis bronchial epithelial cells (CFBE) with dysfunction of the chloride transport.

**Results:**

PM exposure significantly increased ROS synthesis and amplified IL-6 and TNF-α release, particularly in HBE ΔαBK_Ca_ and CFBE cells. Mitochondrial function was also adversely affected, as evidenced by reduced maximal respiratory capacity in both HBE ΔαBK_Ca_ and CFBE cells relative to HBE WT. In addition, PM-treated HBE ΔαBK_Ca_ and CFBE cells showed higher intracellular calcium concentrations. Finally, PM exposure resulted in a pronounced reduction in transepithelial electrical resistance (TEER), with CFBE monolayers exhibiting the most significant susceptibility to barrier disruption.

**Conclusion:**

These findings indicate that impaired potassium and chloride transport through the BK_Ca_ and CFTR channels exacerbates particulate matter–induced oxidative stress, inflammatory responses, mitochondrial dysfunction, and disturbances in calcium homeostasis in airway epithelial cells. Increased susceptibility of HBE ΔαBK_Ca_ and CFBE cells to PM exposure, underscores the crucial role of proper ion transport in maintaining airway epithelial integrity.

## Introduction

Airborne particulate matter (PM) is a major contributor to premature mortality and cardiopulmonary diseases, driving the progression and exacerbation of conditions such as asthma, chronic obstructive pulmonary disease (COPD), idiopathic pulmonary fibrosis, and lung cancer.^1^ We have previously used the commercially available Standard Reference Materials (SRM) from the National Institute of Standards and Technology (NIST) to study effects of the cellular DNA damage response in human bronchial epithelial cells exposed to particulate matter, suggesting a previously unrecognized mechanism by which PM contributes to genomic instability.^2^ Similarly, exposure to polystyrene nanoparticles disrupts DNA repair mechanisms in Caco-2 cells.^3^

PM exposure leads to irreversible cellular and subcellular disruption. The primary target of atmospheric particulate matter is mitochondria, which are crucial organelles of cellular metabolism as key regulators of the electron transport chain. Recent studies suggest that exposure to PM leads to mitochondrial dysfunction and damage.^4^,^5^ At the cellular level, PM can induce oxidative stress and the production of reactive oxygen species (ROS), leading to an imbalance in cellular homeostasis and damage to mitochondria.^6^,^7^ PM_2.5_ (PM size diameter ≤ 2.5 μm) exposure leads to increased production of proinflammatory cytokines such as interleukin-1β (IL-1β), interleukin-6 (IL-6), tumor necrosis factor-α (TNF-α), and interleukin-8 (IL-8).^8–10^ Moreover, ROS activates key signaling pathways such as nuclear factor kappa B (NF-κB) and mitogen-activated protein kinases (MAPKs), which regulate the expression of proinflammatory cytokines.^11^ The activation of the NF-κB pathway is a common mechanism through which PM_2.5_ induces inflammation. This pathway is activated by oxidative stress and leads to the transcription of various inflammatory genes. These inflammatory mediators recruit immune cells to the site of PM deposition, amplifying the local inflammatory response and contributing to tissue damage. The bronchial epithelium forms the first line of defense against inhaled pollutants and acts as a barrier, preventing deeper penetration of harmful agents into the lungs. It is a suitable model of target tissue for PM exposure, as exposure to PMs, especially PM_2.5_, leads to loss of epithelial barrier function and tight junction dysfunction, thereby increasing harmfulness and biological effects.^12^ Human bronchial epithelial (HBE) cells are essential for maintaining the structural and functional integrity of the respiratory tract.^13^,^14^ This protective role is facilitated by their ability to regulate the composition and volume of the airway surface liquid (ASL), a function critical for effective mucociliary clearance and pathogen removal.

ASL homeostasis is a highly complex regulatory process essential for proper lung function and depends on the coordinated transport of ions, solutes, and water through the epithelial barrier.^15^ Because of their high concentrations and membrane conductances, sodium (Na⁺) and chloride (Cl^−^) are the main ions responsible for ASL volume regulation. In contrast, potassium (K⁺) and bicarbonate (HCO_3_^−^), although physiologically significant, are likely to play a regulatory role given their lower concentrations in ASL. Ion transport is mediated by a diverse array of proteins, including sodium (Na⁺), chloride (Cl^−^), and potassium (K⁺) channels.^15^ While Na⁺ and Cl^−^ transport—particularly via ENaC and cystic fibrosis transmembrane conductance regulator (CFTR)—has been extensively studied in lung physiology, the role of K⁺ channels in pulmonary epithelia remains less explored. Nearly 40 K⁺ channel types have been identified in airway and alveolar epithelial cells, many with still-unknown functions. These channels help regulate membrane potential and ion transport and may also contribute to oxygen sensing and mucosal defense, highlighting their emerging importance in lung epithelial physiology.^16^ CFTR plays a pivotal role in chloride transport and ASL hydration. CFTR is a cAMP-regulated apical chloride channel whose dysfunction disrupts epithelial fluid secretion, leading to mucus thickening.^17^ Although the CFTR protein is primarily found in the plasma membrane, there is mounting evidence that it is also present in intracellular organelles, including mitochondria, lysosomes, endosomes, and phagosomes.^17^ It has been demonstrated that CF cells produce more ROS, have higher NOX expression and activity, and have lower intracellular and extracellular glutathione levels.^18^ In addition to abnormal ROS production and associated lipid membrane peroxidation, CFTR mutations affect mitochondria by altering oxygen consumption, lowering mitochondrial membrane potential (ΔΨ), affecting ADP/ATP exchange, and altering complex I and IV activity.^19^ Conversely, the large-conductance calcium-activated potassium channel (BK_Ca_) is crucial for K^+^ ion transport. It modulates potassium efflux, which is essential for setting up the membrane potential and regulating other ion transport activities, including those required for Cl^−^ secretion. BK_Ca_ channels play a role in various cellular processes, including the regulation of oxidative stress and inflammation. It has previously been shown that BK_Ca_ plays a key role in regulating airway surface liquid (ASL) volume in human airways.^20^ Effective transepithelial anion secretion depends on coordinated conductances, including basolateral K⁺ channels that maintain the driving force for Cl^−^ efflux beyond the CFTR channel. It was previously shown that activation of basolateral potassium channels enhances Cl^−^ secretion in human bronchial epithelial cells. At the apical membrane, BK_Ca_ channels also promote Cl^−^ secretion and regulate airway surface liquid (ASL) volume; their inhibition or downregulation by CF-associated inflammatory mediators reduces ASL. These findings suggest that pharmacological activation of BK_Ca_ may offer therapeutic benefit not only in CF,^21^ but also in other airway diseases such as COPD and asthma.^22^ However, the specific role of BK_Ca_ channels in the context of human bronchial epithelial (HBE) cells exposed to PM has not been extensively studied. Interestingly, it was recently demonstrated that the absence of the BK_Ca_ channel (HBE ΔαBK_Ca_ cells) led to a compromised epithelial barrier function, as evidenced by reduced monolayer resistance.^23^ There is evidence that potassium channels, including mitochondrial BK_Ca_ (mitoBK_Ca_), are involved in cytoprotection.^24^ In the current study, human bronchial epithelial cells: HBE WT (wild-type human bronchial epithelial cells), HBE ΔαBK_Ca_ (HBE WT cells with disruption of the gene encoding α-subunit of the BK_Ca_ channel), and CFBE (cystic fibrosis human bronchial epithelial, HBE cells with dysfunction of the chloride transport) cells were exposed to PM to reveal the potential mechanism of PM-elicited human bronchial epithelial cell injury in vitro involving oxidative stress and inflammation.

## Materials and Methods

### PM Sample

Particulate matter with a diameter of less than 4 μm (SRM-2786, NIST, Gaithersburg, MD, USA) was used in the current study, as described previously.^2^,^25^,^26^ We employed PM in accordance with the NIST standard to ensure the consistency and repeatability of our biophysical and biochemical studies. This SRM was prepared from particulate matter collected in 2005 from an air filtration system at an exhibition center in Prague and reflects typical urban airborne particles. The material was resuspended, size-fractionated using a UHVS, and then transferred into amber glass bottles (100–140 mg each) with Teflon-lined caps, as described by the manufacturer. According to the Certificate of Analysis for Standard Reference Material^®^ 2786, the sample contains a complex mixture of organic and inorganic constituents. Certified values are provided for selected polycyclic aromatic hydrocarbons (PAHs), polybrominated diphenyl ethers (PBDEs), and trace elements including Cd, Pb, and Hg. Non-certified values are available for nitro-PAHs, additional PAHs, and PBDE congeners, sugars (eg, levoglucosan), polychlorinated dibenzo-p-dioxins and dibenzofurans (PCDD/Fs), hexabromocyclododecane (HBCD) isomers, and further inorganic constituents. The material also includes information on particle size distribution (mean diameter: 2.8 µm).^26^ Stock suspensions at 50 mg/mL were prepared in PBS, bath-sonicated for 30 min to prevent clustering of the PM particles, and stored at 4 °C. Experiments were performed using the freshly prepared stock solution to minimize variation in particulate matter composition. The suspensions were prepared to final concentrations of 10, 50, and 100 μg/mL PM before direct application to the cell culture, as described in.^2^,^5^,^27–29^

### Cell Culture and Treatments

Human bronchial epithelial cells (16HBE14σ−, CFBE41o−) were obtained from Sigma-Aldrich, Inc. (St. Louis, MO, USA) and cultured according to the recommended conditions. The cells were cultured in a MEM medium with 10% FBS, penicillin, and streptomycin (10 mg/mL). Cells were cultured at 37 °C, 5% CO_2,_ and under 95% humidity. Trypsin-EDTA was added to passage cells at 70–80% confluence after the growth medium was changed, in accordance with the guidelines. Olympus Entry Cell Sense software was utilized to view the cells with a CKX53 Olympus inverted microscope. 16HBE14o^−^ is a human bronchial epithelial cell line derived from a 1-year-old male heart–lung transplant donor and immortalized using an origin-of-replication–defective SV40 plasmid (pSVori^−^). According to the supplier’s description (Sigma-Aldrich), these cells retain key features of differentiated bronchial epithelial cells, including cobblestone morphology, cytokeratin expression, formation of functional tight junctions, and polarized ion transport. The HBE ΔαBK_Ca_ cell line used in this study represents our established experimental model and was generated using CRISPR–Cas9 technology, as previously described in accordance with Nature Protocols,^29^ and has been shown to exhibit physiological characteristics, as recently published.^2^,^23^ CFBE41o^−^ is a human bronchial epithelial cell line derived from a cystic fibrosis patient homozygous for the ΔF508 CFTR mutation and immortalized using an origin-of-replication–defective SV40 plasmid (pSVori^−^).^30^ The cells exhibit hallmark ion transport defects associated with cystic fibrosis, including impaired cAMP-dependent chloride transport while retaining calcium-dependent chloride transport. When cultured under appropriate conditions, CFBE41o^−^ cells form tight junctions and establish a polarized epithelial monolayer. Transepithelial tests in Ussing chambers can be conducted with CFBE41o-cells, as they form tight epithelial monolayers and exhibit a deficiency in cAMP-regulated Cl-ion transport. Cells were incubated with PM at 10, 50, and 100 μg/mL for 24 and 48 hours, directly in cell culture medium, as indicated in each experiment. The choice of PM concentrations was based on our previous experiments.^24^,^31^

### Evaluation of Cell Viability

The impact of the PM on the cell viability of 16HBE14σ, ∆αBK_Ca_16HBE14σ, and CFBE cells was assessed using the 3-(4,5-dimethylthiazol-2-yl)-2,5-diphenyltetrazolium bromide (MTT) assay. Cells were seeded into 96-well plates (Nunc, Thermo Fisher Scientific, Waltham, MA, USA) at a density of 5 × 10^4^ cells per well and cultured until full confluence was reached. Subsequently, cells were exposed for 24 hours to PM at concentrations of 10, 30, and 50 μg/mL. After treatment, the culture medium was replaced with 100 μL of fresh medium containing 0.5 mg/mL MTT. Following a 3-hour incubation, 100 μL of 2-propanol (VWR) was added to solubilize the formazan crystals. Absorbance was measured at 570 nm using a Multiskan SkyHigh microplate reader (Thermo Fisher Scientific, Waltham, MA, USA; serial no. 1600500) after 15 minutes. Cell viability was expressed as a percentage relative to untreated control cells. Each condition was analyzed using at least 8 technical replicates. Experiments were performed on independent cell batches and repeated twice.

### Assessment of Reactive Oxygen Species (ROS) Levels

ROS levels were measured as previously described.^2^ Upon reaching 90% confluence, cells were trypsinized and resuspended in a culture medium at 200,000 cells/mL. A total of 20,000 cells per well were seeded into clear 96-well plates and allowed to adhere for 24 hours. The culture medium was then removed, and the cells were rinsed with PBS. Then, they were incubated for 30 minutes with 10 µM 2′,7′-dichlorofluorescin diacetate (H2DCFDA; Thermo Fisher Scientific, Waltham, MA, USA) to assess total ROS levels. Following dye loading, particulate matter (PM) was added at a concentration of 50 µg/mL, and the cells were further incubated for 3 hours. Fluorescence intensity was measured using a Fluoroskan Ascent plate reader (Thermo Fisher Scientific, Waltham, MA, USA) with excitation/emission wavelengths set to 485/520 nm.

### ELISAs

The concentrations of TNF-α and IL-6 in supernatant were measured using corresponding ELISA kits purchased from Thermo Fisher Scientific (Waltham, MA, USA). All standards were set up using proteins provided by the manufacturer. All experiments were conducted on transparent 96-well plate cells using HBE WT, HBE ΔαBK_Ca_, and CFBE cell lines after 24 and 48 hours of incubation with different concentrations of PM (0, 10, 50, and 100 µg/mL). ELISA samples for IL-6 were also incubated with TNF-α. Data shown represent the average of six replicates of each sample; absorptions of samples were compared to the standard curve, corresponding to the obtained concentrations of IL-6 and TNF-α in cell lines according to the manufacturer’s instructions.

### Measurement of Mitochondrial Respiration

Using the Oxygraph‐2K system (Oroboros Instruments, Austria), high-resolution respirometry was used to measure mitochondrial function as described previously.^23^ After harvesting HBE WT, HBE ΔαBK_Ca_, and CFBE cells, they were centrifuged and resuspended in MEM at a concentration of 1×10^6^ cells/mL. For oxygen consumption measurement, the cells were resuspended and placed in the respiratory chambers. Cell lines were treated with 3 µM FCCP to measure the maximal respiration rate and 4 µg/mL oligomycin to detect a non-phosphorylating leak. 1 μM antimycin A and 1 μM rotenone were added as a last step to assess any remaining oxygen consumption.

### Calcium Flux Assay

To assess calcium levels, live Ca^2+^ imaging using Fura-2-AM (Thermo Fisher Scientific, Waltham, MA, USA) was performed according to the manufacturer’s instructions.^32^ HBE WT, HBE ΔαBK_Ca,_ and CFBE cells were seeded in a 96-well plate. After 24 hours, cells were washed with PBS and replaced with fresh medium. Fura-2-AM was added to the cells to give a final concentration of 3 μM, kept for 45 minutes in an incubator, and then washed free of excess probe. Subsequently, the tested modifying factors (10, 50, and 100 μg/mL PM) and ionomycin in concentration of 5 μM were added in HBSS buffer or in Ringer solution deprived of [Ca^2+^] and with addition of EGTA Fluorescence intensity was measured using a microplate reader, Fluoroskan Ascent (Thermo Fisher Scientific, Waltham, MA, USA) at an excitation wavelength of 340 and 380 nm and an emission wavelength of 538 nm. Results were calculated as the fluorescent ratio of *F*_340_/*F*_380_.^33^

### Transepithelial Electrical Resistance Measurements

The Corning Costar Snapwell inserts were used to seed the cells for transepithelial electrical resistance assessment, as demonstrated in detail earlier.^23^ The EVOM2 voltohmmeter was used to measure the cell layer’s resistance. Each reading was calculated by subtracting the resistance of the empty insert (cell-free Snapwell) and multiplying the result by the surface area of the cell layer. This enabled analysis of the monolayer resistance.

### Statistical Analysis

All experiments were performed in three independent biological replicates to confirm reproducibility. Results were displayed as mean ± SEM by Prism 8 (GraphPad Software Inc). A one-way ANOVA, paired or unpaired *t*-test was used to analyze experimental data. *P*-values were considered significant: *p ≤ 0.05, **p ≤ 0.01, ***p ≤ 0.001.

## Results

This study investigates whether potassium and chloride transport dysfunction could influence the harmful effects of particulate matter on airway epithelial cells. Specifically, the research aimed to assess the impact of PM on oxidative stress, inflammation, mitochondrial function, intracellular calcium balance, and epithelial barrier integrity. Three different airway epithelial cell lines: wild-type human bronchial epithelial cells (HBE WT) with proper function of potassium and chloride transport, and, respectively, with dysfunction of potassium and chloride transport, HBE cells with disruption of the gene encoding the α-subunit of the BK_Ca_ channel (HBE ΔαBK_Ca_), and cystic fibrosis bronchial epithelial cells (CFBE) with the homozygous F508del-*CFTR* mutation.

### PM Depleted Cell Viability in Airway Epithelia

**Figure 1:**
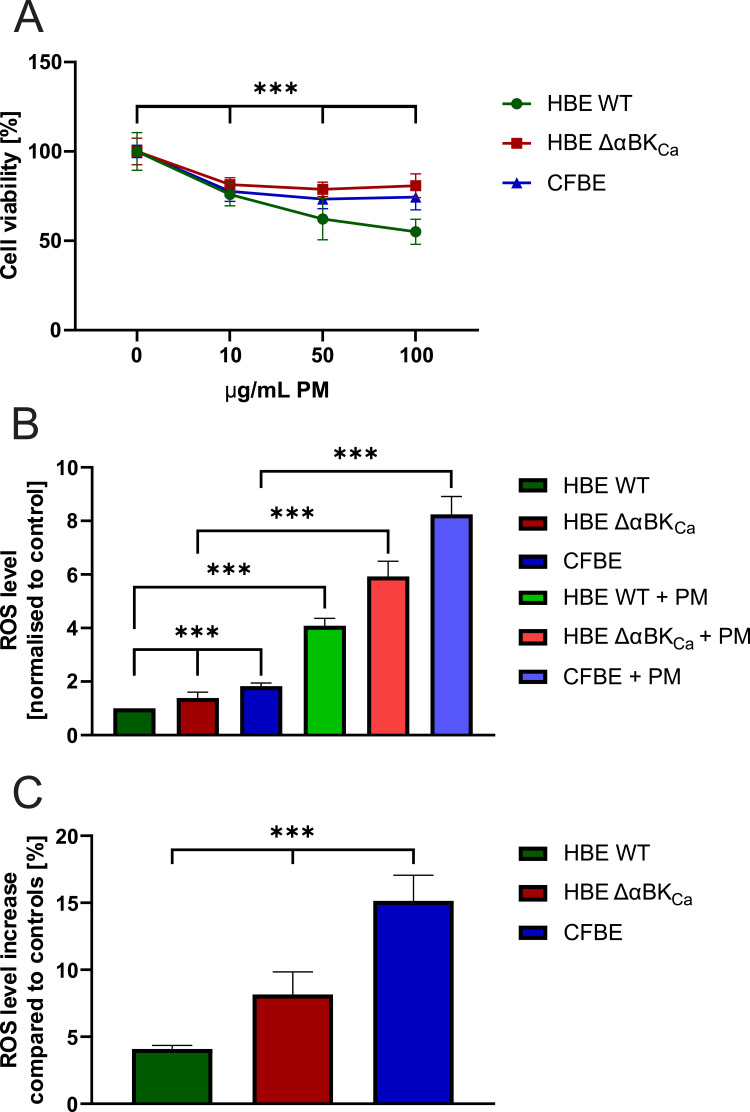
Cell viability and intracellular reactive oxygen species (ROS) level. (**A**) Effect of PM on HBE WT, HBE ΔαBK_Ca_, and CFBE cells in MTT assay. Cells were incubated with PM for 24 hours at concentrations of 10, 50, and 100 µg/mL. Data are presented as mean and SD (n=16). Statistical significance was determined using an unpaired *t*-test (***p<0.001) compared to each cell line control. (**B**) The impact of 50 µg/mL PM exposure on intracellular ROS level (in arbitrary units) in HBE WT, HBE ΔαBK_Ca_, and CFBE cells was presented after 3 hours of incubation. Data were normalised to the control and expressed as mean ± SEM (n=6) for ROS level analysis (H2DCFDA probe). (**C**) Data were expressed as a percentage of intracellular ROS level increase after PM exposure compared to control, % ± SEM (n=6). Statistical significance was determined using one-way ANOVA (***p<0.001).

Evaluation of PM’s potential cytotoxicity was performed using the MTT assay. HBE WT, HBE ΔαBK_Ca_, and CFBE cells were treated with PM for 24 hours, which resulted in a statistically significant decrease in cell viability in all tested concentrations (Figure 1A). Interestingly, 10 μg/mL of PM caused the same level of changes in all investigated cell lines. In HBE ΔαBK_Ca_ and CFBE, effects were stabilized at 10 μg/mL and higher concentrations. Contrary to HBE WT cells, which were most susceptible to PM treatment with concentration-dependent depletion of cell viability, going below other cell lines, with 62 ±7% of living cells for 50 μg/mL of PM and reaching 55 ± 6% for 100 μg/mL.

### PM Exposure Induced Increased ROS Generation in Airway Epithelial Cells

The results of intracellular ROS analysis determined by H2DCFDA fluorescence are shown in Figure 1A and B. Basal ROS levels (Figure 1A) were higher in both HBE ΔαBK_Ca_ and CFBE cells compared to HBE WT, indicating elevated oxidative stress under control conditions. Upon PM exposure (50 µg/mL, 3 h), intracellular ROS levels increased significantly in all cell lines, as measured by absolute fluorescence values normalized to control (Figure 1A, ***p ≤ 0.001). Post-PM ROS levels reached 4.09 ± 0.27 [a.u.] in HBE WT, 5.93 ± 0.56 [a.u.] in HBE ΔαBK_Ca_, and 8.24 ± 0.69 [a.u.] in CFBE cells. To facilitate comparison of PM responsiveness across cell lines, relative changes in ROS levels (% of control) are presented in Figure 1B. PM exposure resulted in a 4.09 ± 0.28% increase in HBE WT cells, an 8.16 ± 1.69% increase in HBE ΔαBK_Ca_ cells, and the largest increase in CFBE cells (15.15 ± 1.90%, ***p ≤ 0.001).

### PM Exposure Induced an Increase in the Production of Proinflammatory IL-6 in Airway Epithelial Cells

ROS generation is linked to the production of inflammatory cytokines, which are crucial mediators of inflammatory reactions. As previously stated, exposure to PM considerably raised the ROS levels in airway epithelial cells. Thus, we treated cells with PM (10, 50, and 100 µg/mL for 24 and 48 hours) to assess the expression levels of proinflammatory cytokines such as interleukin-6 (IL-6). The analysis of IL-6 levels after particulate matter exposure revealed significant differences in the inflammatory response between HBE WT and HBE ΔαBK_Ca_ cells, suggesting a potential impact of the α-subunit deletion on cytokine production. While both cell types exhibited a dose-dependent increase in IL-6 secretion, the magnitude and statistical significance of the response differed.

**Figure 2:**
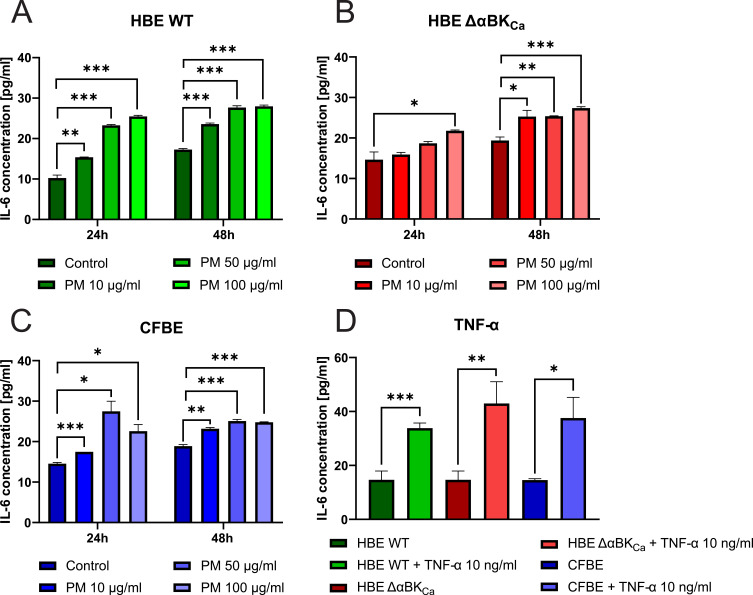
Analysis of the Interleukin-6 (IL-6) presence. IL-6 concentration in HBE WT (**A**), HBE ΔαBK_Ca_ (**B**), and CFBE (**C**) cell lines after 24 and 48 hours of incubation with 10, 50, 100 µg/mL PM (**A**–**C**) and 10 ng/mL TNF-α (**D**). Absorbance results were converted to concentration values. Error bars represent the mean ± SEM (n=3). Statistical significance was determined using one-way ANOVA (*p<0.05, **p<0.005, ***p<0.001) compared to the controls.

At the 24-hour time point, IL-6 levels in HBE WT control cells (Figure 2A) were measured at 10.24 ± 0.98 pg/mL, with exposure to 10 μg/mL PM increasing to 15.39 ± 0.07 pg/mL (**p ≤ 0.01), 50 μg/mL to 23.27 ± 0.27 pg/mL (***p ≤ 0.001), and 100 μg/mL to 25.47 ± 0.33 pg/mL (***p ≤ 0.001). In contrast, HBE ΔαBK_Ca_ cells (Figure 2B) exhibited higher basal IL-6 levels of 14.65 ± 2.48 pg/mL, with exposure to 10 μg/mL PM leading to an increase to 15.90 ± 0.65 pg/mL, 50 μg/mL to 18.67 ± 0.6 pg/mL, and 100 μg/mL to 21.77 ± 0.27 pg/mL (*p ≤ 0.05). While both cell types responded with an increase in IL-6 production, the HBE ΔαBK_Ca_ cells showed a more moderate elevation compared to the WT cells. At the 48-hour time point, a similar pattern was observed. IL-6 levels in HBE WT cells increased from 17.27 ± 0.33 pg/mL in the control group to 23.57 ± 0.33 pg/mL at 10 μg/mL PM (***p ≤ 0.001), 27.67 ± 0.6 pg/mL at 50 μg/mL (***p ≤ 0.001), and 27.97 ± 0.4 pg/mL at 100 μg/mL (***p ≤ 0.001). HBE Δα BK cells exhibited a baseline IL-6 level of 19.37 ± 1.13 pg/mL, increasing significantly to 25.27 ± 1.8 pg/mL at 10 μg/mL PM (*p ≤ 0.05), 25.37 ± 0.13 pg/mL at 50 μg/mL (**p ≤ 0.01), and 27.37 ± 0.4 pg/mL at 100 μg/mL (**p ≤ 0.01). The differences observed between the cell lines suggest that the deletion of the α-subunit may influence the extent and dynamics of the inflammatory response. To further explore the effects of PM exposure on airway epithelial cells, IL-6 levels were measured in CFBE cells at 24 and 48 hours post-exposure (Figure 2C). The results reveal a significant increase in IL-6 secretion with increasing PM concentrations, suggesting a dose-dependent inflammatory response. After 24 hours, the baseline IL-6 level in CFBE control cells was 14.56 ± 0.36 pg/mL, which increased significantly to 17.47 pg/mL upon exposure to 10 μg/mL PM (*** *p* ≤ 0.001). A more pronounced increase was observed at 50 μg/mL PM, reaching 27.47 ± 3.33 pg/mL (*p ≤ 0.05), while exposure to 100 μg/mL PM led to a concentration of 22.57 ± 2.2 pg/mL (*p ≤ 0.05). These data indicate a peak response at 50 μg/mL, followed by a slight reduction at the highest concentration, which may indicate a regulatory or saturation effect. After 48 hours, CFBE control cells exhibited an IL-6 level of 18.87 ± 0.53 pg/mL, which increased significantly to 23.17 ± 0.4 pg/mL after exposure to 10 μg/mL PM (**p ≤ 0.01). Exposure to 50 μg/mL PM resulted in an IL-6 level of 25.07 ± 0.53 pg/mL (***p ≤ 0.001), while the highest PM concentration of 100 μg/mL led to 24.77 ± 0.13 pg/mL (***p ≤ 0.001). These findings suggest a sustained inflammatory response over time, with the 50 μg/mL concentration producing the highest IL-6 levels, consistent with the 24-hour trend. Comparing CFBE cells to HBE WT and HBE ΔαBK_Ca_ cells, CFBE cells exhibited an intermediate inflammatory response. While the CFBE cells produced higher baseline IL-6 levels compared to HBE WT, their response to PM exposure was less pronounced than in HBE ΔαBK_Ca_ cells. The results in Figure 2D demonstrate significant differences in IL-6 secretion across the tested cell lines following exposure to TNF-α (10 ng/mL). The analysis reveals that TNF-α stimulation leads to a substantial increase in IL-6 levels compared to the untreated control conditions, highlighting the pro-inflammatory effects of TNF-α. For HBE WT cells, the mean IL-6 level under control conditions was 10.24 ± 0.98 pg/mL, which increased significantly to 33.82 ± 1.35 pg/mL following TNF-α stimulation, indicating a marked pro-inflammatory response (***p ≤ 0.001). This increase underscores the sensitivity of HBE WT cells to TNF-α-induced IL-6 production. Similarly, HBE ΔαBK_Ca_ cells, which exhibited a baseline IL-6 level of 14.65 ± 5.7 pg/mL, responded to TNF-α exposure with a significant increase to 42.97 ± 5.7 pg/mL, demonstrating an even greater response compared to HBE WT cells (**p ≤ 0.01). This suggests that the absence of the α-subunit may enhance the IL-6 response to TNF-α stimulation. In the case of CFBE cells, the baseline IL-6 level was 14.56 ± 0.36 pg/mL, and TNF-α stimulation increased to 37.57 ± 5.4 pg/mL (*p ≤ 0.05), indicating a notable inflammatory response.

### TNF-α as a Key Proinflammatory Mediator in PM-Exposed Airway Epithelial Cells

**Figure 3:**
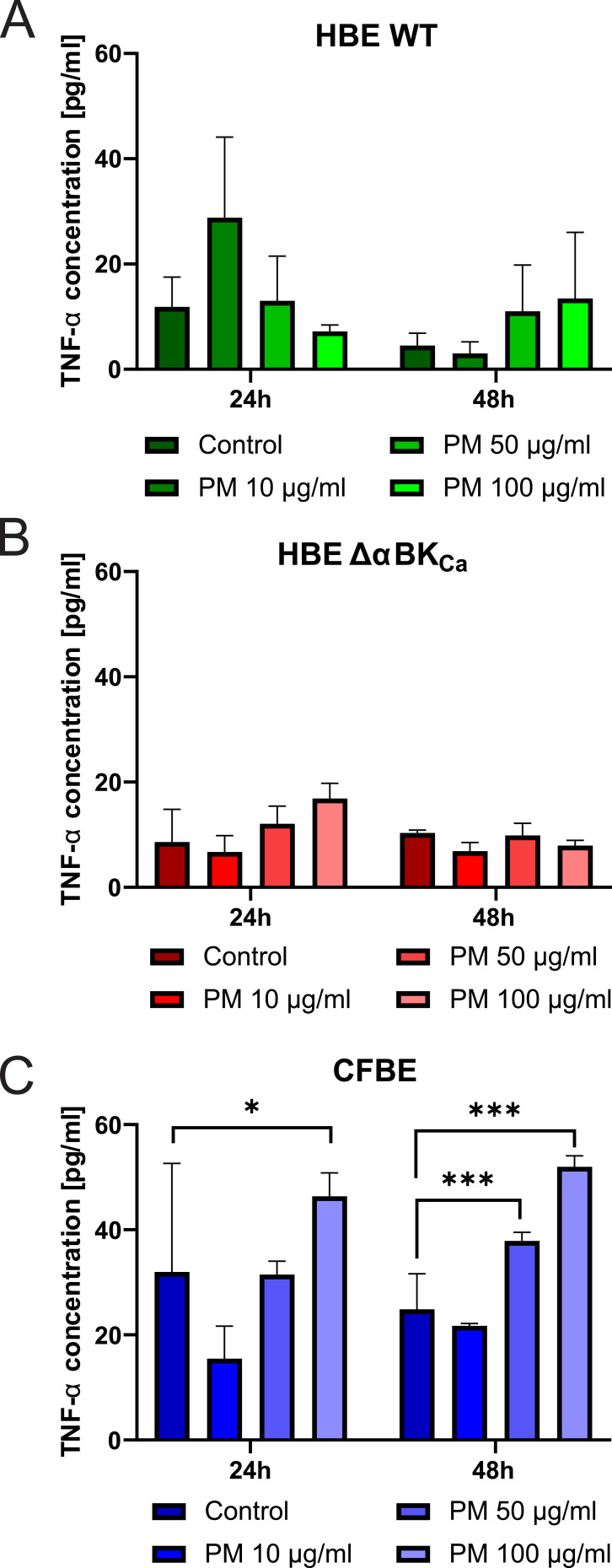
Evaluation of the Tumor Necrosis Factor-α (TNF-α) level. TNF-α concentration in HBE WT (**A**), HBE ΔαBK_Ca_ (**B**), and CFBE (**C**) cell lines after 24 and 48 hours of incubation with 10, 50, 100 µg/mL PM. Absorbance results were converted to concentration values. Error bars represent the mean ± SEM (n=3). Statistical significance was determined using one-way ANOVA (*p<0.05, ***p<0.001) compared to the controls.

TNF-α is a pivotal proinflammatory cytokine that mediates immune responses and coordinates the inflammatory cascade. In our experiments, PM exposure induced distinct TNF-α secretion profiles among the three cell lines (HBE WT, HBE ΔαBK_Ca_, and CFBE) (Figure 3). The TNF-α levels in HBE WT and HBE ΔαBK_Ca_ cells were analyzed at 24-hour and 48-hour time points following exposure to various concentrations of particulate matter. The results demonstrate notable differences in TNF-α secretion between the two cell types, with HBE ΔαBK_Ca_ cells exhibiting lower baseline cytokine levels and a more variable response to PM exposure.

At 24 hours, the baseline TNF-α level (0 μg/mL) in HBE WT cells (Figure 3A) was 11.85 ± 4.01 pg/mL, while HBE Δα BK cells (Figure 3B) showed a lower baseline level of 8.60 ± 4.39 pg/mL, suggesting reduced basal TNF-α production in the absence of the α-subunit. Following exposure to 10 μg/mL PM, TNF-α levels in HBE WT cells significantly increased to 28.82 ± 10.83 pg/mL, whereas in HBE ΔαBK_Ca_ cells, the response was more modest, reaching 6.74 ± 2.17 pg/mL, indicating a reduced sensitivity to lower PM concentrations in the ΔαBK_Ca_ variant. At the 50 μg/mL PM concentration, TNF-α levels in HBE WT cells were 13.02 ± 6.00 pg/mL, compared to 12.08 ± 2.36 pg/mL in HBE ΔαBK_Ca_ cells, suggesting a comparable response at this concentration. However, at the highest dose of 100 μg/mL PM, HBE WT cells exhibited a mean TNF-α level of 7.87 ± 1.21 pg/mL, whereas HBE ΔαBK_Ca_ cells had a significantly higher TNF-α concentration of 16.85 ± 2.07 pg/mL, indicating a stronger late response in ΔαBK_Ca_ cells at higher PM concentrations. At 48 hours, the baseline TNF-α level in HBE WT cells (0 μg/mL) was measured at 4.53 ± 1.64 pg/mL. In contrast, HBE ΔαBK_Ca_ cells maintained a slightly higher basal level of 10.34 ± 0.36 pg/mL, suggesting persistent TNF-α production over time in ΔαBK_Ca_ cells. Exposure to 10 μg/mL PM resulted in TNF-α levels of 3.02 ± 1.57 pg/mL in HBE WT cells, while HBE ΔαBK_Ca_ cells exhibited a higher response at 6.85 ± 1.19 pg/mL, indicating that HBE ΔαBK_Ca_ cells maintain a more sustained inflammatory response over time. At 50 μg/mL PM, TNF-α levels were 11.04 ± 6.21 pg/mL in HBE WT cells and 9.88 ± 1.63 pg/mL in HBE ΔαBK_Ca_ cells, reflecting a comparable response across both cell lines. Interestingly, at 100 μg/mL PM, HBE WT cells showed relatively low TNF-α levels of 7.54 ± 6.85 pg/mL, whereas HBE ΔαBK_Ca_ cells exhibited 7.90 ± 0.73 pg/mL, indicating a more stable response in ΔαBK_Ca_ cells compared to the variability observed in HBE WT cells.

At the 24-hour time point, CFBE cells exhibited a higher TNF-α response at 100 μg/mL PM (46.37 ± 5.11 pg/mL) (Figure 3C) compared to HBE WT (7.87 ± 1.21 pg/mL) and HBE ΔαBK_Ca_ (16.85 ± 2.07 pg/mL), indicating a more robust inflammatory reaction in CFBE cells. The response to 50 μg/mL PM was also significantly higher in CFBE cells (31.50 ± 3.18 pg/mL) than in HBE WT (13.02 ± 6.00 pg/mL) and HBE ΔαBK_Ca_ (12.08 ± 2.36 pg/mL), suggesting greater sensitivity to PM exposure. At the 48-hour time point, TNF-α levels in CFBE cells at 100 μg/mL PM (51.95 ± 2.79 pg/mL) remained significantly higher than in HBE WT (7.54 ± 6.85 pg/mL) and HBE Δα BK_Ca_ (7.90 ± 0.73 pg/mL), showing a sustained inflammatory response. Interestingly, HBE ΔαBK_Ca_ cells demonstrated a prolonged TNF-α secretion at lower PM concentrations, with 6.85 ± 1.19 pg/mL at 10 μg/mL PM, which was significantly higher than the corresponding response in HBE WT cells (3.02 ± 1.57 pg/mL).

CFBE cells displayed the highest TNF-α secretion across all PM concentrations and time points, with a statistically significant increase compared to HBE WT and HBE ΔαBK _Ca_ cells. HBE WT cells exhibited an initial peak response at 10 μg/mL PM that declined at higher concentrations, whereas HBE ΔαBK_Ca_ cells showed a more sustained but moderate response, particularly at lower PM doses.

### Analysis of Mitochondrial Respiration Under TNF-α–Stimulated Conditions

**Figure 4:**
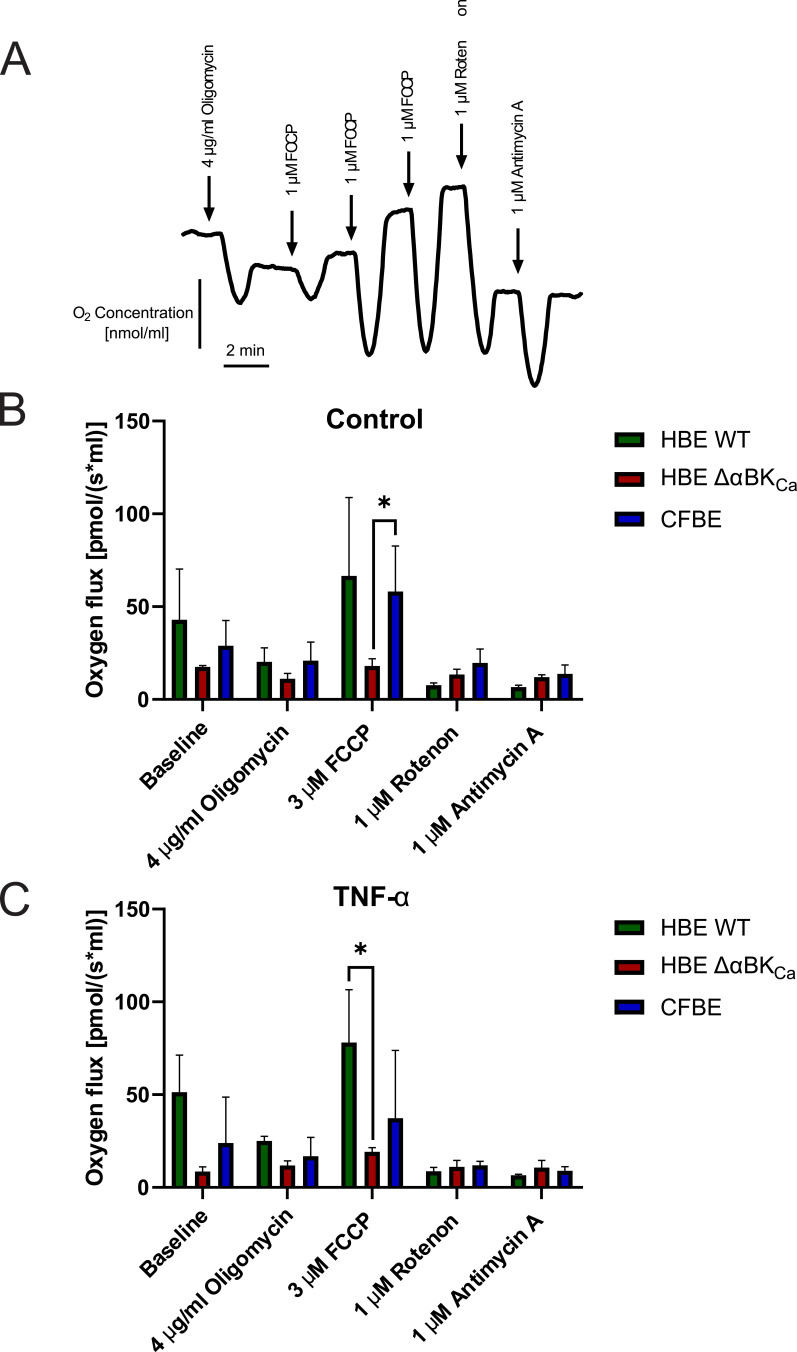
Comparison of mitochondrial oxygen consumption rate in HBE WT, HBE Δα BK_Ca_, and CFBE cells. (**A**) Representative recording of changes in oxygen concentration in HBE WT cells treated with oligomycin (4 µg/mL), FCCP (3 µM), rotenone (1 µM) and antimycin (1 µM). (**B**) Oxygen consumption rate in untreated cells (control). (**C**) Oxygen consumption rate in treated cells after 24 hours of incubation with 10 ng/mL TNF-α, cells were exposed to oligomycin (4 µg/mL), FCCP (3 µM), rotenone (1 µM), and antimycin A (1 µM). Results were obtained using a probe measuring the OCR, and shown as mean ± SEM (n=3). Statistical significance was determined using one-way ANOVA (*p<0.05) compared to each cell line.

We aimed to determine whether BK_Ca_ channel disruption (HBE ΔαBK_Ca_) and a CF background (CFBE) affect mitochondrial function and how TNF-α might further modulate this response. A compromised or aberrant mitochondrial respiration rate, measured by measuring oxygen consumption, is the main indicator of mitochondrial dysfunction.^23^ Representative traces of oxygen concentration over time during a mitochondrial respiration assay are presented in Figure 4A. Sequential additions of inhibitors and uncouplers were performed to assess different aspects of mitochondrial function. We added 4 μg/mL of oligomycin, an inhibitor of mitochondrial ATP synthase, to assess non-phosphorylating proton leaks. At a concentration of 3 μM, the protonophore FCCP was used to measure the maximum mitochondrial respiration rate. Furthermore, respiratory chain complexes I and III were inhibited with rotenone (1 μM) and antimycin A (5 μM), respectively, to measure non-mitochondrial oxygen consumption. As shown in Figure 4A and B, at baseline, HBE WT cells had the highest respiration rate (42.89 ± 21.12 pmol/(s*mL)) compared to HBE ΔαBK_Ca_ (17.52 ± 0.61 pmol/(s*mL)) and CFBE (28.91 ± 10.52 pmol/(s*mL)). After exposure to 4 μg/mL oligomycin, respiration decreased in all cell types, with HBE Δα BK_Ca_ cells showing the most significant decrease (11.19 ± 2.23 pmol/(s*mL)). With 3 μM FCCP, a mitochondrial uncoupler, all cell types showed increased respiration, with HBE WT showing the largest increase (66.54 ± 32.47 pmol/(s*mL)). CFBE cells showed 58.14 ± 18.21 pmol/(s*mL) and HBE ΔαBK_Ca_ cells had 18.06 ± 2.96 pmol/(s*mL), which was statistically significantly higher than HBE ΔαBK_Ca_ (*p ≤ 0.05). Following 1 μM rotenone exposure, all cells showed a decrease, with HBE ΔαBK_Ca_ showing the lowest reduction (13.41 ± 2.15 pmol/(s*mL)). After TNF-α stimulation (Figure 4C), HBE WT cells exhibited the highest baseline oxygen flux (51.25 ± 15.34 pmol/(s*mL)), while HBE ΔαBK_Ca_ cells showed lower values (8.53 ± 1.96 pmol/(s*mL)). Following mitochondrial inhibition with 4 μg/mL oligomycin, oxygen flux decreased in HBE WT cells to 25.06 ± 1.76 pmol/(s*mL), with HBE ΔαBK_Ca_ cells showing the lowest levels (11.85 ± 1.87 pmol/(s*mL)). After exposure to 3 μM FCCP, oxygen flux increased in all cell types, with HBE WT cells showing the highest levels (78.11 ± 21.79 pmol/(s*mL)). In HBE ΔαBKₐ, oxygen flux increased to 19.19 ± 1.59 pmol/(s*mL), and in CFBE to 37.26 ± 28.10 pmol/(s*mL). HBE WT cells exhibited a significantly higher oxygen flux compared to HBE ΔαBK_Ca_ (*p ≤ 0.05). Following exposure to 1 μM rotenone, oxygen flux decreased across all cell lines, with HBE ΔαBK_Ca_ showing the lowest values (11.04 ± 2.52 pmol/(s*mL)). CFBE cells showed a flux of 11.97 ± 1.49 pmol/(s*mL), while HBE WT cells exhibited 8.67 ± 1.57 pmol/(s*mL).

### PM-Induced Dysregulation of Intracellular Ca^2^⁺ Levels in Airway Epithelium Cells

**Figure 5:**
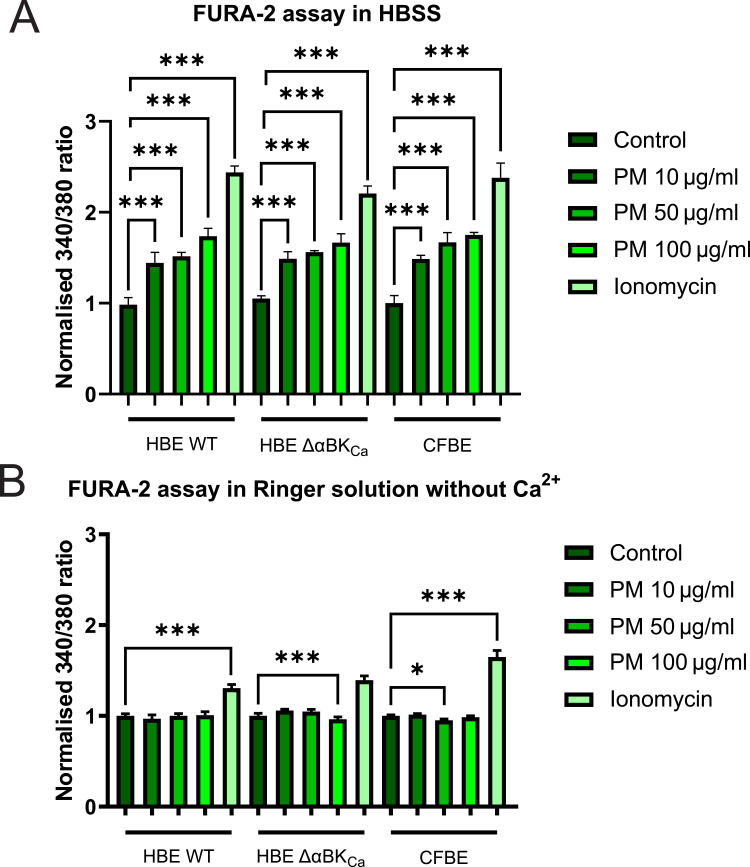
Analysis of the calcium intracellular content. (**A**) Representation of the intracellular Ca^2+^ levels in HBE WT, HBE Δα BK_Ca_, and CFBE cell lines in HBSS buffer. (**B**) Representation of the intracellular Ca^2+^ levels in HBE WT, HBE Δα BK_Ca_, and CFBE cell lines in Ringer solution deprived of Ca^2+^ and EGTA addition. Cell lines were treated with 10, 50, and 100 µg/mL PM and 5 µM ionomycin (positive control). Results were calculated as the fluorescent ratio of *F*_340_/*F*_380_ and presented as mean ± SD (n=7). Statistical significance was determined using an unpaired *t*-test (*p<0.05, ***p<0.001) compared to each cell line.

We next investigated whether PM-induced oxidative stress also perturbs intracellular calcium homeostasis in HBE WT, HBE ΔαBK_Ca_, and CFBE cells by measuring changes in [Ca^2^⁺] using the Fura-2 ratiometric dye. As shown in Figure 5A, exposure to increasing concentrations of PM (10, 50, and 100 µg/mL) in HBSS buffer led to a significant, concentration-dependent elevation of intracellular Ca^2^⁺ in all three cell lines compared to controls. Ionomycin treatment (calcium ionophore), included as a positive control, produced the most significant increase in intracellular Ca^2^⁺, confirming the responsiveness of all cell lines to maximal calcium influx. For the PM 10 μg/mL condition, HBE WT cells exhibited calcium levels of 1.44 ± 0.08 [a.u.], which was significantly higher than the control (***p ≤ 0.001). HBE ΔαBK_Ca_ and CFBE cells showed slightly higher values of 1.49 ± 0.06 [a.u.] and 1.49 ± 0.02 [a.u.], respectively, with both showing a significant increase compared to the control (***p ≤ 0.001). The differences between the cell types at this concentration are minimal, with CFBE cells demonstrating the lowest variability. Under the PM 50 μg/mL condition, calcium levels increased across all cell types. HBE WT cells reached 1.52 ± 0.03 [a.u.], showing a significant increase compared to the control (***p ≤ 0.001). HBE ΔαBK_Ca_ cells increased to 1.56 ± 0.01 [a.u.], and CFBE cells exhibited the highest levels at 1.67 ± 0.08 [a.u.], both of which were significantly higher than their respective control values (***p ≤ 0.001). In response to PM 100 μg/mL, calcium levels were comparable in HBE WT cells (1.74 ± 0.06 [a.u.]), slightly exceeding the levels observed in CFBE cells (1.75 ± 0.02 [a.u.]), with both showing significant increases compared to control values (***p ≤ 0.001). HBE ΔαBK_Ca_ cells showed a relatively lower response (1.67 ± 0.07 [a.u.]), which was still significantly higher than the control (***p ≤ 0.001), suggesting a potential regulatory effect in calcium handling under higher PM exposure.

To assess whether the increase in calcium level was caused by the release of intracellular [Ca^2+^] stores or by extracellular influx, we performed another set of experiments in calcium-free buffer. As shown in Figure 5B, calcium levels in WT HBE, HBE ΔαBKCa, and CFBE cells stimulated by 10, 50, and 100 µg/mL of PM in Ringer solution deprived of [Ca2+] and with the addition of EGTA are presented. Results clearly show no effect of an increase in [Ca^2+^] in FURA-2-loaded cells, indicating that elevated cellular calcium content was caused by extracellular influx.

### PM Exposure Reduced Epithelial Barrier Integrity

**Figure 6:**
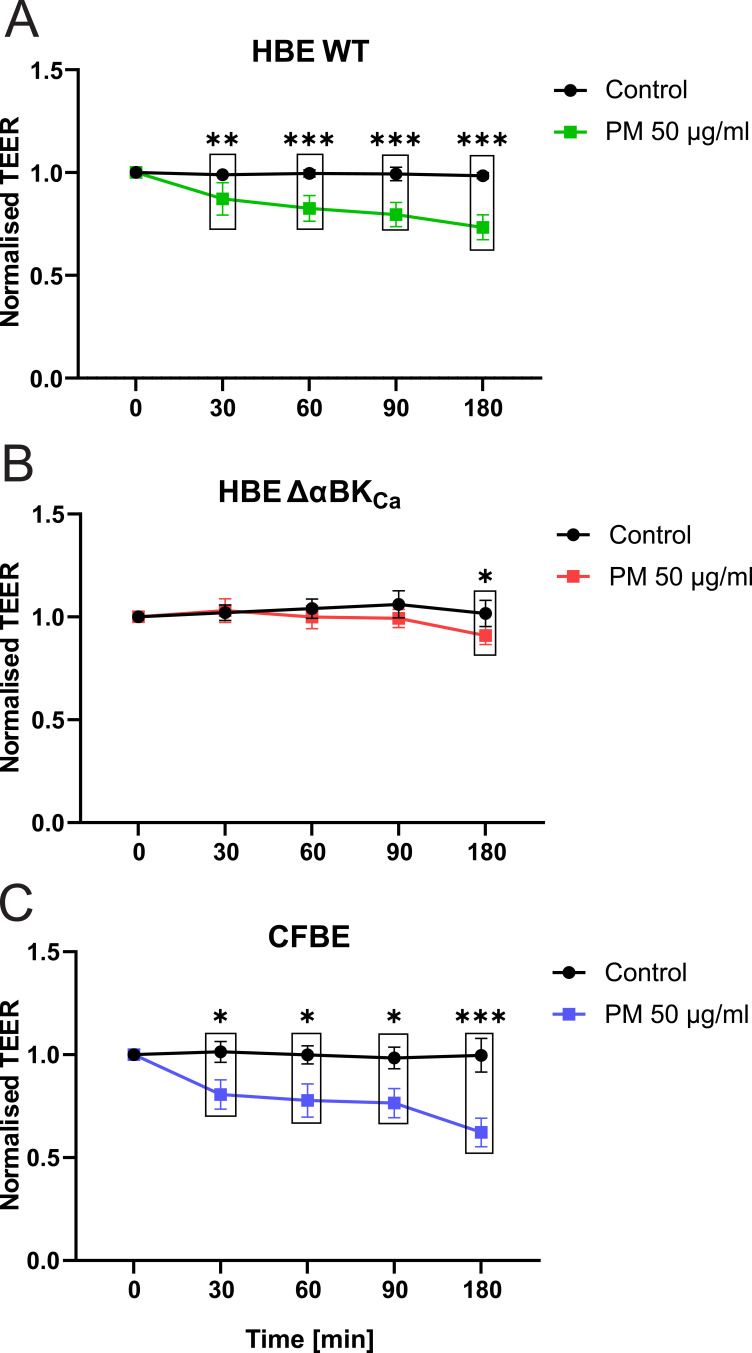
Changes in transepithelial electrical resistance (TEER) on airway epithelium in the presence of particulate matter. (**A**) Impact of PM on TEER in HBE WT cell monolayers. (**B**) Effect of PM on HBE ΔαBK_Ca_ cell monolayers. (**C**) Impact of particulate matter on transepithelial electrical resistance in CFBE cell monolayers. Cell monolayers were treated with 50 µg/mL PM, and TEER was measured after 30, 60, 90, and 180 minutes of incubation. Results were normalised to the TEER value measured before treatment (time 0 min) and were shown as mean values ± SD (n=6). Statistical significance was determined using a paired *t*-test (*p<0.05, **p<0.01, ***p<0.001) compared to each cell line.

Given the pronounced oxidative stress, inflammatory responses, and calcium dysregulation observed in earlier experiments, we next determined whether these effects translate into compromised epithelial barrier function by measuring transepithelial electrical resistance (TEER) (Figure 6A–C) for HBE WT, HBE ΔαBK_Ca,_ and CFBE cell lines. In the HBE cells, exposure to PM 50 μg/mL led to a progressive decline in TEER values [Ω·cm^2^ normalised to cell line controls], starting from 1.00 ± 0.00 at baseline to 0.73 ± 0.02 [a.u.] after 180 minutes (***p ≤ 0.001), indicating a significant disruption of the epithelial barrier over time. In the CFBE cells, cells exposed to PM 50 μg/mL experienced a substantial decrease in TEER over time, with values declining from 1.00 ± 0.00 [a.u.] at baseline to 0.62 ± 0.14 [a.u.] after 180 minutes (***p ≤ 0.001), suggesting a more pronounced loss of barrier integrity compared to HBE cells. The HBE ΔαBKCa cell line showed the least pronounced reduction in barrier integrity, with TEER values decreasing to 0.91 ± 0.04 after 180 minutes of PM exposure (*p ≤ 0.05).

## Discussion

Particulate matter exposure induces oxidative stress, critical in triggering inflammatory responses in airway epithelial cells. PM leads to the generation of reactive oxygen species during the phagocytosis of particles by alveolar macrophages and other inflammatory cells, amplifying oxidative stress and inflammation.^34^ This oxidative stress activates redox-sensitive signalling pathways, producing pro-inflammatory cytokines such as TNF-α and IL-6, which were elevated in the studied cell types, particularly in CFBE cells. The interaction between ROS and inflammatory mediators creates a feedback loop, where ROS-induced inflammation generates further ROS, exacerbating the overall inflammatory response.^35^,^36^

The observed mitochondrial dysfunction in HBE ΔαBK_Ca_ and CFBE cells under TNF-α stimulation highlights the role of mitochondria as a main source of ROS. PM-induced disruption of mitochondrial electron transfer exacerbates ROS production and mitochondrial damage, further contributing to oxidative stress and inflammation.^37^ Moreover, potassium channels in the inner mitochondrial membrane, such as mitoBK_Ca_, are critical for cytoprotection by modulating mitochondrial function and ROS levels. The dysfunction of BK_Ca_ channels in HBE ΔαBK_Ca_ cells might explain their heightened susceptibility to PM-induced stress, aligning with previous findings.^24^

Alongside oxidative and inflammatory processes, the BK_Ca_ and CFTR channels are key regulators of potassium (K^+^) and chloride (Cl^−^) transport, essential for preserving the balance of ASL and the integrity of epithelial barriers.^38^ BK_Ca_ channels are involved in K^+^ efflux, setting the membrane potential in epithelial cells, which influences the function of other ion channels, notably CFTR. CFTR is primarily responsible for Cl^−^ secretion across the apical membrane, which is the primary mechanism that transports water into the airway lumen, ensuring the hydration of the ASL. In cystic fibrosis, mutations in the CFTR gene impair Cl^−^ transport, resulting in thick, dehydrated mucus and compromised mucociliary clearance. These conditions are also linked to increased mitochondrial reactive oxygen species production and heightened inflammatory signaling, as demonstrated in cystic fibrosis bronchial epithelial cells exposed to particulate matter. Additionally, the lack of functional BK_Ca_ channels, as seen in human bronchial epithelial cells without the BK_Ca_ α subunit, disrupts potassium homeostasis, potentially weakening mitochondrial defense against oxidative stress. Together, these mechanisms highlight the critical role of K^+^ and Cl^−^ transport in protecting epithelial cells against environmental pollutants, such as particulate matter.

PM is a known factor that negatively affects cell viability; for example, HBE cells exhibit decreased proliferation.^39–41^ The CFBE cells were also examined, and it was proven that PM stimulation led to increased apoptosis.^42^ Data in the literature is coherent, stating cytotoxicity of PM, but no publication was made to compare the effect of PM on different airway cell lines, with the exception of one work where HBE ΔαBK_Ca_ and HBE WT were stimulated by PM in a clonogenic assay,^2^ loss of functional BK_Ca_ channel resulted in fewer colonies formed in comparison to WT. In our MTT assay results, the most susceptible cell line to PM treatment was HBE WT. This phenomenon could be explained by conducting different types of experiments on two aspects: proliferation and cytotoxicity. Perhaps, cell lines with disrupted functional channels are characterised by greater resistance to stressors like PM, in exchange for reduced proliferation potential and other health markers.

Additionally, PM exposure disrupted intracellular calcium homeostasis and reduced epithelial barrier integrity, as evidenced by decreased transepithelial electrical resistance. These effects are consistent with prior studies linking oxidative stress to calcium dysregulation and impaired epithelial function, further emphasizing the synergistic impact of oxidative stress and inflammation on airway damage.^43^,^44^ The most resistant cell line to PM treatment in terms of epithelial integrity was HBE ΔαBK_Ca_. This effect could be attributed to unstimulated, already low TEER values, approximately halved relative to HBE WT,^33^ and significantly decreased prior to PM application. These findings underscore the central role of oxidative stress in mediating the cellular and inflammatory responses to PM exposure, particularly in cells with predisposing conditions such as CFTR or BK_Ca_ channel dysfunction. The interdependence of ROS generation, mitochondrial dysfunction, and inflammatory signalling highlights potential therapeutic targets, including mitochondrial protection and modulation of redox-sensitive pathways.

Chronic exposure to environmental pollutants drives the development and progression of diseases such as asthma and chronic obstructive pulmonary disease (COPD). Inhaled pollutants disrupt the airway epithelial barrier, leading to inflammation, airway remodelling, and increased susceptibility to respiratory infections, thereby promoting disease exacerbations and progression. Understanding how inhaled pollutants contribute to these diseases is essential for elucidating their pathogenesis and for informing prevention and treatment strategies.

Our results suggest that cellular responses to particulate matter (PM) exposure are associated with the electrophysiological properties of the cells studied, indicating that targeting specific ion channels may offer clinical benefits. Although there is currently no cure for chronic respiratory diseases, new therapeutic approaches are needed. It is well established that PM exposure increases airway epithelial permeability, elevates reactive oxygen species (ROS) production, and triggers inflammatory responses.^45^,^46^ However, our findings demonstrate that PM-induced effects are linked to the activity of specific ion channels, suggesting that modulation of these channels may provide protection against PM exposure. One potential strategy involves the use of naturally occurring, plant-derived secondary metabolites that have been shown to enhance epithelial barrier integrity, reduce ROS production, and attenuate inflammatory responses.^47^,^48^ Importantly, these compounds have also been reported to modulate the ion channel activities.^48^ However, given the complex and multifactorial nature of airway disease pathogenesis, single-agent therapies are often insufficient. This underscores the need for combination treatment strategies and suggests that flavonoids may serve as adjuncts to existing therapies for airway diseases rather than standalone treatments.

Although this study offers important insights into airway epithelial responses to PM exposure, several limitations warrant consideration. First, the experiments were conducted using widely used bronchial epithelial cell models (16HBE14σ−, CFBE41o−), as well as a 16HBE14o− cell line with disrupted BK_Ca_ channel activity. While these models are well established, they may not fully recapitulate the physiology of primary human bronchial epithelial cells. Future studies using primary cells and fully differentiated epithelial models are needed to better investigate the effects of PM exposure, particularly with respect to mucociliary clearance and the complexity of innate immune responses.

Second, this study employed a standardized PM sample, which may not accurately represent real-world air pollution. Ambient PM composition varies seasonally and geographically, posing challenges for accurately reflecting true personal exposure across different populations and environments worldwide. Additionally, our experiments did not investigate the long-term effects of PM exposure.

## Conclusions

In conclusion, alterations in potassium and chloride transport significantly exacerbate cellular damage and indicate the importance of BK_Ca_ and CFTR channels in maintaining epithelial integrity. Furthermore, these results may aid in developing strategies to protect the airway epithelium, which is at increased risk due to impaired ion transport or environmental stress. Additionally, we provide a solid foundation for future research to identify factors that increase epithelial susceptibility to particulate matter exposure.

It appears that future research should focus on discovering strategies to preserve or restore BK_Ca_ and CFTR activity, thereby enhancing cellular defense against particulate matter-induced damage. Although these findings provide valuable insights into epithelial susceptibility to particulate matter in vitro, further in vivo and clinical studies are necessary to determine their significance under physiological exposure conditions.

## Data Availability

The data used and/or analysed during the current study are available from the corresponding author on reasonable request.

## Abbreviations

BK_Ca_, Large Conductance Calcium-Activated Potassium Channel; CF, Cystic Fibrosis; CFBE, Cystic Fibrosis Human Bronchial Epithelial cells; CFTR, Cystic Fibrosis Transmembrane Conductance Regulator; COPD, Chronic Obstructive Pulmonary Disease; CRISPR-Cas9, Clustered Regularly Interspaced Short Palindromic Repeats–CRISPR-associated protein 9; ELISA, Enzyme-Linked Immunosorbent Assay; FCCP, Carbonyl cyanide-4-(trifluoromethoxy)phenylhydrazone (a mitochondrial uncoupler); Fura-2-AM, Cell-permeant fluorescent calcium indicator dye; H2DCFDA, 2′,7′-Dichlorofluorescein diacetate (a fluorescent probe for ROS measurement); IL-1β, Interleukin-1β; IL-6, Interleukin-6; IL-8, Interleukin-8; MAPKs, Mitogen-Activated Protein Kinases; NF-κB, Nuclear Factor kappa-light-chain-enhancer of activated B cells; PM, Particulate Matter; ROS, Reactive Oxygen Species; TEER, Transepithelial Electrical Resistance; TNF-α, Tumor Necrosis Factor-α.
